# The Cellular Response to Oxidatively Induced DNA Damage and Polymorphism of Some DNA Repair Genes Associated with Clinicopathological Features of Bladder Cancer

**DOI:** 10.1155/2016/5710403

**Published:** 2015-11-16

**Authors:** Nataliya V. Savina, Nataliya V. Nikitchenko, Tatyana D. Kuzhir, Alexander I. Rolevich, Sergei A. Krasny, Roza I. Goncharova

**Affiliations:** ^1^Institute of Genetics and Cytology, National Academy of Sciences of Belarus, 27 Akademicheskaya Street, 220072 Minsk, Belarus; ^2^N.N. Alexandrov National Cancer Centre of Belarus, Lesnoy, 223040 Minsk, Belarus

## Abstract

Genome instability and impaired DNA repair are hallmarks of carcinogenesis. The study was aimed at evaluating the DNA damage response in H_2_O_2_-treated lymphocytes using the alkaline comet assay in bladder cancer (BC) patients as compared to clinically healthy controls, elderly persons, and individuals with chronic inflammations. Polymorphism in DNA repair genes involved in nucleotide excision repair (NER) and base excision repair (BER) was studied using the PCR-RFLP method in the Belarusian population to elucidate the possible association of their variations with both bladder cancer risk and clinicopathological features of tumors. The increased level of H_2_O_2_-induced DNA damage and a higher proportion of individuals sensitive to oxidative stress were found among BC patients as compared to other groups under study. Heterozygosity in the *XPD* gene (codon 751) increased cancer risk: OR (95% CI) = 1.36 (1.03–1.81), *p* = 0.031. The frequency of the *XPD* 312Asn allele was significantly higher in T ≥ 2 high grade than in T ≥ 2 low grade tumors (*p* = 0.036); the *ERCC6* 1097Val/Val genotype was strongly associated with muscle-invasive tumors. Combinations of homozygous wild type alleles occurred with the increased frequency in patients with non-muscle-invasive tumors suggesting that the maintenance of normal DNA repair activity may prevent cancer progression.

## 1. Introduction

Oxidized DNA base lesions induced by environmental pollutants and endogenous metabolites lead to a variety of mutations and consequently to genetic instability, which is a hallmark of cancer [1, 2]. As shown in European populations, increased frequencies of chromosome aberrations and micronuclei are closely associated with cancer risk [3, 4]. When monitoring genomic alterations in the urothelial carcinomas in individual patients for a long time, the increased level of the mitotic recombination was found at the early carcinogenesis stage, and extensive genetic damage was accumulated during the evolution of the tumors [5]. When studying the cellular response to DNA damage in different types of cancer, activation of the ATM–Chk2–p53 signal pathway was observed in early human tumorigenesis [6] indirectly indicating accumulation of DNA lesions that, in turn, might be considered as the primary trait of upcoming genome instability and cell malignancy. Thus, the oxidatively induced DNA damage initiating genome instability is one of the principal factors of carcinogenesis, whereas the other one seems to be DNA repair deficiency or impairment.

The oxidatively damaged bases are predominantly removed via the BER pathway initiated with their excision by DNA glycosylases [7]. Among them, 8-oxo-guanine DNA glycosylase 1 (OGG1) is responsible for elimination of the highly mutagenic DNA lesion, 8-oxo-7,8-dihydroguanine (8-oxoGua). Another functionally important protein, X-ray repair cross-complementing protein 1 (XRCC1), interacts with DNA glycosylases, AP endonuclease-1 (APE-1), DNA polymerase *β* (POL*β*), DNA ligase III (Lig III), poly (ADP-ribose) polymerase 1 (PARP-1), and polynucleotide kinase (PNK) at the damaged site, by modulating their activities and coordinating the subsequent enzymatic BER steps [8, 9]. The reduced BER activity has been newly discussed to trigger the development of sporadic cancers [10]. The performed proteomic analysis of BER deficient human cells has demonstrated that BER deficiency, leading to genome instability, results in dramatic changes in gene expression, resembling changes found in many cancers. These findings suggest that genetically unstable BER deficient cells may be a source of precancerous cells [10].

The majority of chemically induced DNA adducts are removed by the NER pathway that operates globally throughout the genome (global genome, GG NER) or during transcription (transcription coupled, TC NER); both subsets differ only in their initial recognition of the helix-distorting DNA damage [11, 12]. In this multistep repair process, DNA helicases unwind the double helix, thus opening access to the lesion site for other repair enzymes [13]. The XPD helicase, mutated in the cancer-prone xeroderma pigmentosum (XP), is part of the TFIIH complex that is essential for signaling events triggering transcription, cell cycle checkpoints, and DNA repair [14]. TC NER requires specific factors, including Cockayne syndrome (CS) protein B (CSB). The latter belongs to both the helicase superfamily 2 and to the SWI/SNF complex maintaining and remodeling chromatin structure [15, 16] and it acts at the crossroads of transcriptional networks [17, 18]. In the context of the present study, the recently reported data confirming involvement of NER-initiating proteins in the elimination of oxidatively generated DNA damage [19, 20] take on special significance.

We attempted to estimate the cellular response to oxidatively induced DNA damage and polymorphism in some DNA repair genes in bladder cancer. The frequencies of* OGG1* Ser326Cys (rs1052133),* XRCC1* Arg399Gln (rs25487),* XPD* Asp312Asn (rs1799793), and* ERCC6* Met1097Val (rs2228526) polymorphisms have been recently determined in the bladder cancer (BC) patients as compared with clinically healthy residents of Belarus [21]. Our results indicated the association of the* XPD* 312Asp/Asn heterozygous genotype with an increased risk of bladder cancer, whereas the* OGG1* 326Ser/Cys heterozygous genotype has exhibited the protective effect. Here, genome integrity and stability was analyzed in peripheral blood lymphocytes using the comet assay. Besides, isolated DNA samples were genotyped for polymorphism of DNA repair genes involved in BER and NER to elucidate both the possible impact of some other genetic variations (*XPD* Lys751Gln and* ERCC6* Gly399Asp) on bladder cancer susceptibility and the association of all six polymorphisms with clinicopathological parameters of tumors for evaluating their prognostic relevance.

## 2. Materials and Methods

### 2.1. Study Populations

The study included two independent experimental sets. In the first experimental set, the control group comprised 35 clinically healthy volunteers aged 22–63 years old who had no chronic and acute diseases and contacts with occupational hazards. They were recruited among residents of regions that were not affected by the Chernobyl fallout. Forty individuals with histologically verified bladder cancer (BC) were randomly selected among patients of the Department of Urology of N.N. Alexandrov National Cancer Centre of Belarus in 2011. The average age of BC patients was about 70 years; males and smokers amounted to 85% and 89% of the sample, respectively. Besides, the group of elderly people was represented by fifteen clinically healthy persons over 60 years; among them, 73% were males. Fifteen individuals comprised the group of chronic inflammatory diseases including chronic obstructive pulmonary disease (9 cases), chronic pyelonephritis (4 cases), and rheumatic disease and polyarthritis (2 cases); all of them were beyond the exacerbation phase. Their average age was about 50 years, and males amounted to 53% of the sample. The cellular response to oxidatively induced DNA damage was compared in these four groups using the comet assay.

In the second experimental set, the case group comprised 418 BC patients who were treated at the Department of Urology of N.N. Alexandrov National Cancer Centre of Belarus over 2011–2014. All urothelial carcinoma diagnoses were verified histologically after transurethral resection of tumors. The T stages were determined using the international Tumor-Node-Metastases (TNM) classification, and the grade of tumor tissue differentiation was established according to WHO classifications of 1973 and 2004 [22, 23]. Blood samples (3–5 mL) were collected by venal puncture by the qualified medical personnel in accordance with the Declaration of Helsinki (1964) [24]. The blood samples were accompanied with a demographic profile of patients and the clinicopathological description of tumors (Table 1).

370 individuals were randomly recruited as controls among healthy volunteers involved in blood donation at the Republic Research and Production Center for Transfusiology and Medical Biotechnologies (Minsk) and elderly people who were observed at the Department of Gerontology and Geriatrics at the Belarusian Medical Academy of Postgraduate Education. Individuals from both control subgroups had no positive cancer history or acute diseases and should be considered the population-based controls. The control population was predominantly represented by males (68.7%). Like the BC patients, noncancer individuals were between 31 and 94 years old, with the average age of 64.5 ± 13.5 years as opposed to 66.7 ± 10.9 years in the case group. The controls were matched to the cases by the recruitment period, the ethnic origin (both were predominantly Belarusians or other Eastern Slavs), and age. However, they differed from each other in the smoking status, since smokers amounted to 31% among controls and to 68% among patients. It should be mentioned that the same control population was used in the previous work in order to study the possible impact of* OGG1* Ser326Cys (rs1052133),* XRCC1* Arg399Gln (rs25487),* XPD* Asp312Asn (rs1799793), and* ERCC6* Met1097Val (rs2228526) polymorphisms on susceptibility to bladder cancer [21]. Herein, the control population was used for the similar purpose concerning* XPD* Lys751Gln (rs13181) and* ERCC6* Gly399Asp (rs2228528) polymorphisms, while the next steps of the study were carried out using the enlarged case group stratified into several categories depending on tumor stages and grades.

Informed consent was obtained from each participant included in the study before the collection of blood samples. All participants were interviewed to complete a questionnaire covering medical, residential, and occupational history as well as age, gender, and the tobacco smoking status. The smoking status was summarized as “smokers” (combining current smokers and ex-smokers) or “nonsmokers” (including never smoking persons).

### 2.2. Estimation of Genome Integrity in Freshly Isolated Lymphocytes Using the Comet Assay

The approach for evaluation of genome integrity in order to diagnose genome instability in isolated lymphocytes was earlier described in detail [25–27]. In this investigation, 2-3 mL peripheral blood was collected into the heparinized Vacutainer tubes and kept at 4°C for no longer than 2 h. Lymphocytes were isolated from whole blood samples by centrifugation over 2.5 mL Histopaque at 1500 rpm for 30 min. Then lymphocytes were washed twice with RPMI 1640, suspended in cold PBS, and exposed to hydrogen peroxide (100 *μ*M H_2_O_2_) at 4°C for 1 min, followed by washing with cold PBS. Intact and treated cells were incubated in RPMI 1640 with 10% fetal bovine serum (FBS) during a 3-h period at 37°C. Their viability was traditionally evaluated with the trypan blue exclusion test and usually varied in the range of 96–98%.

All the reagents and procedures were used according to the admitted protocol of the alkaline comet assay (single cell gel electrophoresis) [28]. Briefly, procedures included slide preparation, lysis of cell membranes for DNA elution by keeping the slides in the cold lysing solution (2.5 M NaCl, 10 mM Na_2_EDTA, 10 mM Tris, 1% Triton-X100, pH 10) for 1 h, DNA unwinding in fresh electrophoresis buffer (1 mM Na_2_EDTA, 300 mM NaOH) for 20 min, and horizontal electrophoresis for 20 min at 1 V/cm, 300 mA and pH > 13. After electrophoresis, slides were washed twice for 5 min with 0.4 M Tris buffer (pH 7.5) for neutralization and fixed in ice-cold 96% ethyl alcohol for 10 min. After staining with ethidium bromide, slides were analyzed with a fluorescence microscope Olympus BX-50. Visual estimation of DNA damage in arbitrary unites (a.u.) was carried out according to published recommendations [29]. Two slides were prepared for each point of analysis, and at least 100 cells were scored per each of two replicate slides by one researcher that provided the concordance between the results. The levels of DNA damage were calculated as average values.

Basal DNA damage was determined after 180 min incubation of intact lymphocytes in RPMI 1640 with 10% FBS. The initial level of oxidatively induced DNA damage was estimated immediately after mutagenic treatment, and the residual level of DNA damage was measured 180 min after exposure. To estimate DNA repair kinetics, samples of H_2_O_2_-treated lymphocytes were collected at 0, 30, 60, and 180 min of their incubation.

### 2.3. Genotyping

DNA for genotyping procedures was extracted using the traditional phenol-chloroform technique. Single nucleotide polymorphisms (SNPs) in some DNA repair genes were determined by the PCR-RFLP method under conditions used in the previous work [21]. In addition to polymorphisms* OGG1* Ser326Cys,* XRCC1* Arg399Gln,* XPD* Asp312Asn, and* ERCC6* Met1097Val,* XPD* Lys751Gln (rs13181) and* ERCC6* Gly399Asp (rs2228528) were analyzed in the present study. These polymorphisms were detected at conditions described elsewhere [30, 31]. The PCR products were digested with restriction enzymes, electrophoresed through 2.5% agarose gels containing ethidium bromide, and visualized under UV light. DNA repair genes and corresponding genotypes are shown in Table 2.

### 2.4. Statistical Analysis

Pearson's *χ* ^2^ test (or Fisher's exact test when necessary) was used to verify the significance of differences between the groups of BC patients and controls as well as between groups of different tumor stage and grade categories in genotype/allele frequencies. Student's *t*-test was used for comparison of the groups by age and other continuous variables, including the DNA damage levels. Nonparametric Mann-Whitney *U* test was also used in the latter case. DNA repair efficiency (RE) was calculated as percentage of DNA lesions eliminated at consequent time points relative to their initial level. The DNA repair rate in different groups was compared by the coefficients of linear regression (*β*) [25–27].

When genotyping the DNA samples for DNA repair gene polymorphisms, the statistical significance for deviation from Hardy-Weinberg equilibrium was determined using *χ* ^2^ test. *p* ≤ 0.05 values were considered significant. The relative risk was estimated as odds ratio (OR) with 95% confidence intervals (CI).

## 3. Results

### 3.1. Genome Integrity In Isolated Lymphocytes after Oxidative Stress* In Vitro*

**Figure 1:**
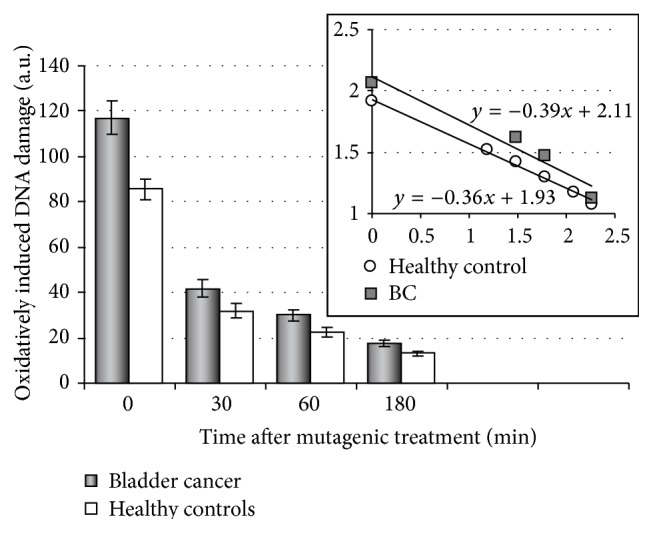
The oxidatively induced DNA damage and DNA repair kinetics in isolated lymphocytes from BC patients as compared to healthy donors. The case group included 40 BC patients; the control group comprised 35 clinically healthy donors. The insert reflects the DNA repair kinetics on a logarithmic scale. The coefficients of linear regression in the groups of patients (*β* = −0.39) and controls (*β* = −0.36) are approximately equal.

To estimate adequately the cellular response to oxidized DNA damage in bladder cancer, it was compared among several groups and first of all between the cases and controls (Table 3, Figure 1). The results indicated the absence of statistically significant differences between the levels of basal DNA damage in the control and case groups, whereas the levels of H_2_O_2_-induced DNA damage in BC patients exceeded those in healthy volunteers during the whole period of observations. The greatest differences were observed immediately after lymphocyte mutagenic exposure. Nevertheless, the slopes of repair kinetics closely resembled each other (the insert in Figure 1), and, being compared by means of regression analysis, these data revealed no differences between two groups with respect to the DNA repair velocity. DNA repair efficiency was also equal in lymphocytes from patients and controls (Table 3), suggesting that induced DNA damage was eliminated in a similar manner in both groups.

Then the cellular response to H_2_O_2_ exposure in BC patients was compared with that in elderly persons and individuals with chronic inflammatory diseases (Table 4). The significant differences were found between all the groups with respect to the initial levels of oxidatively induced DNA damage, with the highest level in the BC patients indicating increased cellular sensitivity to oxidative stress in bladder cancer.

In another approach, the frequency of sensitive individuals (with an enhanced DNA damage response) was estimated in the same groups using the earlier established reference intervals for all the parameters under study in the control population of 172 residents of Belarus [32]. In brief, the normal lymphocyte response to DNA damage was determined due to calculating 10th and 90th percentiles for levels of basal and exogenous DNA damage as well as for DNA repair efficiency measured at certain time points after mutagenic exposure. The marginal values were determined as follows: 15 a.u. for basal DNA damage, 110 a.u. for the initial level, 25 a.u. for the residual level of H_2_O_2_-induced DNA damage, and 70% for DNA repair efficiency by the end of cell incubation. Subjects with the levels of DNA damage exceeding these values as well as with DNA repair efficiency, which is lower than the normal parameter, were attributed to the group of “sensitive” individuals. It is seen from Table 5 that half of the BC patients sample manifested the increased sensitivity of lymphocytes to H_2_O_2_ immediately after treatment as opposed to 14.3% in the control group and 26.7% among elderly persons and individuals with chronic inflammations, respectively. Both approaches have demonstrated that the cellular responses to oxidatively induced DNA damage in bladder cancer strongly differed from those in healthy donors and to a lesser degree in aging and inflammations. Consequently, the increased initial level of H_2_O_2_-induced DNA damage in isolated lymphocytes might serve as a potential biomarker of genome instability predisposing to cancer.

### 3.2. Association of DNA Repair Gene Polymorphisms with Bladder Cancer Risk and Clinicopathological Characteristics of Tumors

Polymorphism in some DNA repair genes has been recently reported to affect susceptibility to bladder cancer in Belarus [21]. Herein, the results of genotyping for* XPD* Lys751Gln (rs13181) and* ERCC6* Gly399Asp (rs2228528) polymorphisms are added (Table 6). The* ERCC6* Gly399Asp polymorphisms were found to be neutral unlike the* XPD* polymorphisms. In the latter case, cancer risk was mainly associated with the* XPD* 751Lys/Gln heterozygous genotype (OR (95% CI) = 1.36 (1.03–1.81) (*p* = 0.031)), which indicated that heterozygosity in this codon predisposes to tumorigenesis as it was earlier noticed for the* XPD* codon 312.

The comparison of the genotype distribution depending on the tumor stages and tumor tissue differentiation (Table 7) revealed lack of differences, except for the* ERCC6* Met1097Val polymorphism (rs2228526). The frequency of the* ERCC6* 1097Val/Val genotype was significantly increased in muscle-invasive tumors as compared to non-muscle-invasive ones (*p* = 0.0045), and the similar trend concerned the Val allele frequency, which was almost doubled in patients with T2 tumors as compared to Ta neoplasms (37.5% and 20.9%, resp.; *p* = 0.0009). These data suggested that the carriers of the* ERCC6 *1097Val allele, predominantly in the homozygous state, have a higher probability of developing advanced cancer, what is also indicated by the odds ratio: OR (95% CI) = 2.86 (1.36–6.05) (*p* = 0.0061) for the Val/Val genotype.

**Figure 2:**
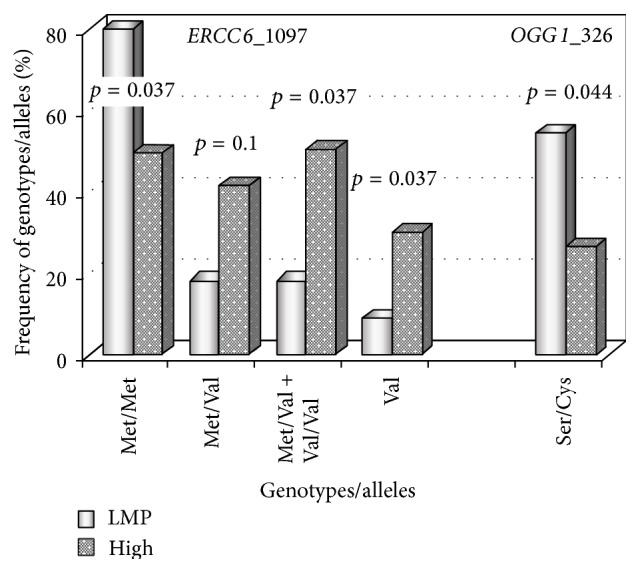
Distribution of some genotypes/alleles in patients with papillary urothelial neoplasms of low malignant potential (LMP, 11 samples) as compared to high grade carcinomas (high, 156 samples). Four coupled bars correspond to* ERCC6* Met1097Val polymorphisms. The frequencies of the Met/Met genotype are 81.8% and 49.4% in LMN and high grade tumors, whereas the frequencies of the Met/Val + Val/Val genotypes are 18.2% and 50.6% in the same types of urothelial carcinomas, respectively. The last coupled bars reflect the frequencies of the* OGG1* (codon 326) heterozygous genotype, which are 54.5% in LMP neoplasms and 26.3% in high grade tumors.

The distribution of genotypes/alleles for polymorphisms of DNA repair genes did not depend on tumor grades. However, the analysis of genetic variations in papillary neoplasms of low malignant potential (PNLMP) as compared with high grade or poorly differentiated cancer revealed some peculiarities concerning* ERCC6* Met1097Val and* OGG1* Ser326Cys polymorphisms (Figure 2). The frequencies of homozygous wild type genotype of* ERCC6* gene and heterozygous genotype of the* OGG1* gene were significantly increased in LMP tumors, whereas the genotypes containing at least one variant allele of the* ERCC6* gene occurred more often in patients with G3 or high grade urothelial carcinomas. Thus, neoplasms of low malignant potential were distinct from others with respect to genotype distribution of both the* OGG1* Ser326Cys and the* ERCC6* Met1097Val polymorphisms.

**Figure 3:**
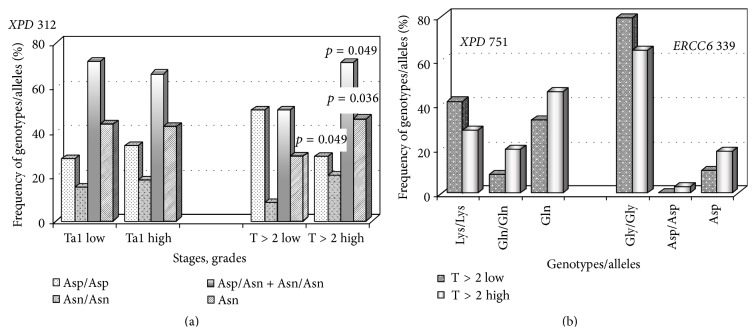
Distribution of some genotypes/alleles of DNA repair genes in non-muscle-invasive and muscle-invasive tumors depending on their grades. (a) The frequencies of* XPD* Asp312Asn polymorphisms in Ta/T1 low grade tumors (227 samples) as compared to Ta/T1 high grade neoplasms (59 samples) as well as in T ≥ 2 low grade tumors (24 samples) as opposed to T ≥ 2 high grade neoplasms (97 samples). In the latter case, the frequencies of the Asp/Asp, Asn/Asn, and Asn/Asn+Asp/Asn genotypes and the Asn alleles were as follows: 50% and 28.9% (*p* = 0.049), 8.3% and 20.6% (*p* = 0.16), 50% and 71.1% (*p* = 0.049), and 29.2% and 45.9% (*p* = 0.036) in T ≥ 2 low grade and T ≥ 2 high grade tumors, respectively. (b) The frequencies of* XPD* (codon 751) and* ERCC6* (codon 399) polymorphisms in T ≥ 2 low grade tumors (24 samples for each polymorphisms) as compared to T ≥ 2 high grade neoplasms (95 and 97 samples, resp.).

When dividing tumors into four categories (Ta/T1 low, Ta/T1 high, T ≥ 2 low, and T ≥ 2 high), evident differences were found only in muscle-invasive carcinomas, with the homozygous wild type genotype of the* XPD* gene (codon 312) being associated with low grade cancer, whereas the frequencies of genotypes containing a variant Asn allele were significantly increased in high grade neoplasms (Figure 3(a)). Two other polymorphisms (*XPD* Lys751Gln and* ERCC6* Gly399Asp) showed similar trends, but the differences between T ≥ 2 low grade tumors and T ≥ 2 high grade carcinomas were not statistically confirmed (Figure 3(b)).

**Figure 4:**
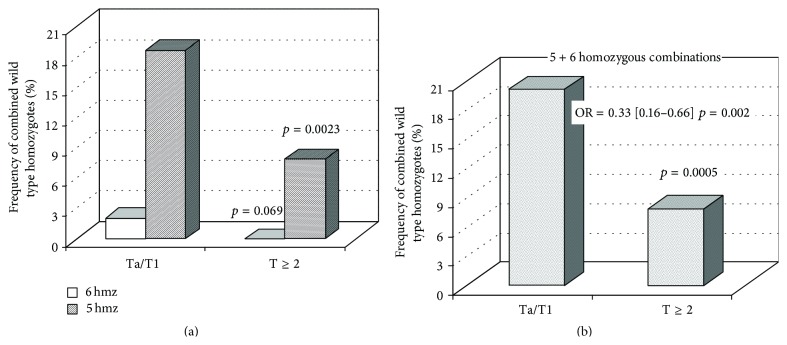
Distribution of wild type homozygous combinations in non-muscle-invasive and muscle-invasive urothelial carcinomas. 6 hmz correspond to combination of all six wild type homozygotes; 5 hmz correspond to total combinations containing any five wild type homozygotes. The Ta/T1 group was represented by 289 samples, whereas the T ≥ 2 group consisted of 120 samples.

Based on the assumption that the wild type of DNA excision repair genes may provide elimination of mutagenic/carcinogenic DNA lesions thus promoting both decrease in cancer risk and inhibition of tumor expansion/malignancy, the frequencies of combined homozygous wild type alleles were estimated depending on T stages. The combination involving Ser/Ser, Arg/Arg, Asp/Asp, Lys/Lys, Met/Met, and Gly/Gly genotypes of* OGG1* (codon 326),* XRCC1* (codon 399),* XPD* (codons 312 and 751), and* ERCC6* (codons 1097 and 399), respectively, was a rare event occurring only in Ta/T1 tumors (2.1%). As shown in Figure 4(a), the total frequency of combinations represented by any five homozygous wild type genotypes was significantly higher in non-muscle-invasive carcinomas (18.7%) as compared to advanced tumors (7.9%) and together with the former combination containing all six wild type homozygotes they achieved 20.8% in Ta/T1 neoplasms as opposed to 7.9% in T ≥ 2 tumors (Figure 4(b)). Accordingly, homozygosity for the wild type alleles of the DNA repair genes under study seemed to prevent tumor expansion. The distribution of these combinations did not depend on tumor grades.

## 4. Discussion

Before discussing the data, it should be noted that evaluation of bladder cancer risk implies a case-control study, but the comparison of the demographic profiles of BC patients and controls (Section 2.1 and Table 1) shows the significant differences between groups concerning tobacco smoking status that is likely to limit interpretation of the results. On the other hand, the random selection of sizeable populations, which are matched by the recruitment period, age and ethnicity, allows a higher frequency of smokers among BC patients to be considered as a disease-specific feature in support of findings that bladder cancer is an age-, gender-, and smoking-related disease [33, 34]. It should also be mentioned that the percentage of smokers in the control population reflects the situation with tobacco consumption in Belarus, and the gene-smoking relationship in bladder cancer has been previously characterized [21, 35]. A close association of bladder cancer with age and a tobacco smoking habit suggests that oxidative stress contributes to its development.

The comet assay used in the first experimental set remains a widespread and efficient tool in biomonitoring studies [36, 37]. Our approach resembles a challenge assay, which is based on detecting chromosome breakage and has developed for revealing exposure-induced DNA repair deficiency as a functional biomarker of cancer risk [38]. In our studies, basal and exogenous DNA lesions were identified as potential biomarkers of genome destabilization, and their levels as well as DNA repair kinetics after oxidative stress* in vitro* were measured as average group values and individually. Using this approach, we diagnosed and specified genome instability in lymphocytes of patients with some genetic disorders and occupationally exposed subjects [25–27]. Taking into account conflicting data on the relationship between age and the yield of DNA damage in the comet assay [36, 39], the age differences, in particular between groups of BC patients and individuals with chronic inflammations (Table 4), might be a limitation of the present study. However, lack of such correlation for basal and H_2_O_2_-induced DNA damage [27] confirms the reliability of our observations.

Herein, the significantly increased levels of H_2_O_2_-induced DNA damage were found in lymphocytes of BC patients as compared to controls with the pronounced effect immediately after mutagenic treatment due to dysfunction of antioxidant defense and disturbance of redox homeostasis [40, 41] rather than a reduced DNA repair rate or efficiency (Figure 1 and Table 3). However, reactive oxygen species (ROS) are known to contribute to inflammations [42, 43], aging, and related diseases [44, 45], whereas inflammations, resulting from and triggering ROS production, forego and accompany carcinogenesis [46–48]. Therefore, it was reasonable for comparing the cellular response to oxidative stress in different conditions. The initial levels of H_2_O_2_-induced DNA damage as well as the proportion of individuals with increased cellular sensitivity to hydrogen peroxide in the group of BC patients exceeded those among elderly persons and subjects with chronic inflammatory diseases (Tables 4 and 5). The results seemed to demonstrate an essential role of the abnormal cellular response to oxidatively generated DNA damage in bladder cancer.

Among various underlying mechanisms, mutations or polymorphisms in genes responsible for antioxidant defense, redox regulation, and oxidatively damaged base repair are currently studied. Our second experimental set was focused on the latter mechanism, and it would be interesting to discuss involvement of DNA helicases in removing oxidized DNA lesions. An inability to repair oxidatively generated damage accumulating in the brain was hypothesized to cause the neurological degeneration in xeroderma pigmentosum [49]. As recently reported, the neurodegeneration in Cockayne syndrome is associated with ROS-induced damage in the mitochondria, independent of nuclear transcription coupled repair [50]. Moreover, CSB protein appears to behave as an electron scavenger in the mitochondria whose absence leads to increased levels of ROS in CSB-mutated cells [50]. The CSA and CSB proteins, in addition to their basic role in TC NER, can participate in BER directly by interaction with BER proteins and indirectly by modulating gene expression [51]. Using high performance liquid chromatography coupled to electrochemical detection (HPLC-EC) to measure the genomic 8-oxoGua levels in mouse NER- or BER-deficient embryo fibroblasts [20] as well as the immunofluorescence method to detect binding of CSB and XPC to oxidative lesions in different nuclear compartments in fibroblast cell lines derived from patients [19], the experimental evidence for a direct involvement of some XP and CS gene products in repair of oxidatively induced damage has been provided. In spite of the fact that the OGG1 DNA glycosylase dominates in 8-oxoGua repair, NER (XPC and XPA) and transcription-coupled repair proteins (CSB and CSA) are similar but are minor contributors [19].

In our studies, the* OGG1* (codon 326) heterozygous genotype decreased bladder cancer risk, especially in smokers with OR = 0.55 (0.34–0.89) (*p* = 0.014) [21] and prevented high grade tumors as compared to neoplasms of low malignant potential (Figure 2). Our findings, at least with respect to cancer predisposition, are in line with some other data [52, 53]. The* ERCC6/CSB *1097Val/Val genotype enhanced susceptibility to advanced (T ≥ 2) urothelial carcinoma (Table 7), and the* ERCC2/XPD* 312Asn allele seemed to promote tumor malignancy, since its frequency was increased in patients with T ≥ 2 high grade tumors as compared to T ≥ 2 low grade neoplasms (Figure 3(a)). The effects associated with impaired activity of CSB and XPD proteins might be mediated by accumulation of ROS, which act as the second messengers in intracellular signaling cascades inducing and maintaining the oncogenic phenotype of cancer cells [54, 55].

It is generally accepted that the “driver” mutations in a few key genes trigger certain (sometimes alternative) pathways of cancer pathogenesis. In bladder cancer, the mutations in* FGFR3* gene are strongly associated with superficial tumors, whereas mutations in* TP53* gene lead to muscle-invasive cancer [56]. However, the molecular analysis of tumor tissue samples from the same Belarusian patients has shown that about 30% among them are of the wild type genotype with respect to both genes suggesting multiple genetic origins of urothelial carcinomas [57]. The current molecular-genetic analysis of bladder cancer includes the whole genome sequencing, detection of genome-wide gene expression profiles, studies of DNA repair and replication processes, the immune and inflammatory responses, and other common hallmarks of human cancers [58–62]. These investigations are aimed at revealing novel molecular markers with high predictive and prognostic relevance. In the context of our study, the results concerning a set of mutations, which were not earlier recognized as significant events for bladder cancer, are of great interest. Specifically, among 32 identified genes, there was the NER* ERCC2/XPD* gene, and its fifteen of sixteen genetic variations were represented by deleterious missense mutations with dominant negative effects [63].

In spite of the fact that genetic variations in excision repair genes are not attributed to driver mutations in bladder cancer [62], they may modulate susceptibility to cancer initiation and cancer progression. For example, genome-wide association studies (GWAS) have identified more than 300 validated associations between genetic variants and risk of approximately 70 common diseases [64]. The functions of genes identified as relevant for bladder cancer focus on detoxification of carcinogens, maintenance of DNA integrity, control of the cell cycle, and apoptosis. Our data indicate both the accumulation of oxidatively induced DNA damage and impact of modified XPD and CSB proteins on risk and clinical course of bladder cancer. It is typical that all known SNPs are associated with bladder cancer with odds ratios lower than 1.5; however, when interacting, they may collectively result in a substantial cancer risk [64]. Combinations of the homozygous wild type alleles are expected to exert a reverse effect. Indeed, the combined wild type homozygotes for some DNA repair genes reduced susceptibility to bladder cancer [35] and even prevented tumor expansion (Figure 4(b)).

The impact of excision repair gene polymorphisms on susceptibility to different cancers, including urothelial carcinoma, has been widely discussed in literature [21, 35]. Their associations with clinicopathological parameters of tumors are still poorly understood, although there are intriguing findings indicating the dual effects of DNA repair gene polymorphisms with respect to bladder cancer risk/recurrences/progression and clinical outcomes. Improved overall and disease-specific survivalas well as decreased mortality risk of BC patients after chemotherapy and radiotherapy was observed in carriers of variant allelesof the* XPC* gene [65] and the* XPD* 751Gln allele combined with the* XPC* 939Gln allele [66]. The clinical outcomes were also affected by a series of* XRCC1* polymorphisms [67] and by the* OGG1* 326 Cys/Cys genotype [68]. Hence, so-called “risky” genotypes/alleles of some DNA repair genes decreased tumor resistance to radiation or chemical treatment, thereby improving clinical outcomes. At the same time, the “risky”* ERCC6* 1097Val allele increased the frequency of urothelial carcinoma recurrences [69] and the* XRCC1* 399 A/A (Gln/Gln) genotype greatly reduced recurrence free survival of BCG treated patients [70]. The higher frequency of muscle-invasive tumors was observed in carriers of* XRCC1* 194 СT+TT genotypes as compared to the wild type CC genotype [70] and in carriers of the mutated* APE1* 148Glu allele [71]. Our results indicating the association of some polymorphic variants of* ERCC6/CSB* and* ERCC2/XPD* genes with advanced bladder cancer (T ≥ 2 as compared to Ta/T1 tumors or T ≥ 2 high as opposed to T ≥ 2 low grade carcinomas) fit into an overall picture, but the problem needs to be further explored to confirm some regularities arising from our own and literature data.

## 5. Conclusion

Using the alkaline comet assay, the increased level of H_2_O_2_-induced DNA damage was found in isolated lymphocytes of BC patients as compared to healthy controls, elderly people, and individuals with chronic inflammatory diseases. The proportion of individuals with the enhanced cellular response to oxidative stress was also significantly higher among BC patients than among healthy subjects. These results showed that accumulation of oxidatively induced DNA damage might serve as a potential biomarker of genome instability predisposing to cancer.

Some excision repair gene polymorphisms modified the susceptibility to bladder cancer and were associated with clinicopathological parameters of tumors. Polymorphisms in* XPD* gene (codons 312 and 751) increased cancer risk, and at the same time the variant* XPD* 312Asn allele was significantly associated with muscle-invasive high grade tumors. Polymorphisms in* ERCC6* gene (codon 1097), especially the Val/Val homozygous genotype, occurred with the higher frequency in muscle-invasive tumors as compared to non-muscle-invasive ones, and polymorphic variants in the* XPD* (codon 751) and* ERCC6* (codon 399) genes manifested the trends resembling effects of the* XPD* Asp312Asn polymorphisms with respect to T ≥ 2 high grade tumors as compared to T ≥ 2 low grade carcinomas. Interestingly, the combinations of homozygous wild type genotypes were associated with non-muscle-invasive tumors and their frequency was more than twice lower in T ≥ 2 carcinomas suggesting that the maintenance of normal DNA repair activity, specifically of some XP and CS gene products, seems to inhibit cancer initiation and/or cancer progression. Based on the literature data, one can assume that their positive effects, at least in part, are mediated through elimination of mutagenic/carcinogenic oxidatively induced DNA damage.

## Figures and Tables

**Table 1:** The demographic features of BC patients and clinicopathological parameters of tumors.

Features	Patients	
	*n*	Frequency %
Gender		
Males	344	82.3
Females	74	17.7
Age (years)		
Min	31	
Max	93	
Mean ± SD		66.7 ± 10.9
Median	67	
Smoking		
Smokers	283	67.7
Nonsmokers	117	28.0
Not specified	18	4.3
Tumor stages		
TIS	1	0.2
Ta	91	21.8
T1	198	47.4
T2	72	17.2
T3	27	6.5
T4	27	6.5
Not specified	2	0.4
Tumor grades		
1973		
CIS	1	0.2
G1	139	33.3
G2	186	44.5
G3	86	20.6
Not specified	6	1.4
2004		
PUNLMP	11	2.6
CIS	1	0.2
Low	241	57.7
High	156	37.3
Not specified	9	2.2
Recurrence		
No	268	64.1
Yes	150	35.9

**Table 2:** Characteristics of allelic variants and some conditions for their detection.

Gene polymorphisms	Primer sequences	Restriction enzyme	PCR products (bp)
Nucleotide excision repair			

*ERCC2/XPD *Asp312Asn rs1799793	(F) 5′-CTG TTG GTG GGT GCC CGT ATC TGT TGG TCT-3′ (R) 5′-TAA TAT CGG GGC TCA CCC TGC AGC ACT TCC T-3′	StyI	Asp/Asp: 507 + 244; Asp/Asn: 507 + 474 + 244 + 33; Asn/Asn: 474 + 244 + 33

*ERCC2/XPD *Lys751Gln rs13181	(F) 5′-GCC CGC TCT GGA TTA TAC G-3′ (R) 5′-CTA TCA TCT CCT GGC CCC C-3′	Pst I	Lys/Lys: 290 + 146; Lys/Gln: 290 + 127 + 146; Gln/Gln: 227 + 146

*ERCC6/CSB *Met1097Val rs2228526	(F) 5′-CCT GCT T CT AAC ATA TCT GT-3′ (R) 5′-AAT CAC TGA CAA CTC TTC TG-3′	Nla III	Met/Met: 123 + 78; Met/Val: 201 + 123 + 78; Val/Val: 201

*ERCC6/CSB *Gly399Asp rs2228528	(F) 5′-TGA AGA GTC TGA GTA TTT CC-3′ (R) 5′-ATC TTC ATC TCC ATC ATC TC-3′	RsaI	Gly/Gly: 180 + 91; Gly/Asp: 271 + 180 + 91; Asp/Asp: 271

Base excision repair			

*XRCC1* Arg399Gln rs25487	(F) 5′-GGA CTG TCA CCG CAT GCG TCG G-3′ (R) 5′-GGC TGG GAC CAC CTG TGT T-3′	MspI	Arg/Arg: 115 + 34; Arg/Gln: 149 + 115 + 34; Gln/Gln: 149

*OGG1* Ser326Cys rs1052133	(F) 5′-CTG TTC AGT GCC GAC CTG CGC CGA-3′ (R) 5′-ATC TTG TTG TGC AAA CTG AC-3′	MboI	Ser/Ser: 224 + 23; Ser/Cys: 247 + 224 + 23; Cys/Cys: 247

**Table 3:** The cellular response to the oxidative stress *in vitro* in the case group as compared to controls.

Features under study	Exposure, time of lymphocyte incubation (min)	BC patients (*n* = 40)	Controls/healthy donors (*n* = 35)
Basal DNA damage	Intact lymphocytes		
	180	11.4 ± 1.0	9.2 ± 0.8

Oxidatively induced DNA damage	Exposure to H_2_O_2 _		
	0	117.1 ± 7.1	85.6 ± 4.4^a^
	30	41.8 ± 3.8	31.7 ± 3.2^a^
	60	29.9 ± 2.7	22.8 ± 2.1^a^
	180	17.7 ± 1.6	13.3 ± 1.1^a^

DNA repair efficiency	30	65.2	63.0
	60	74.5	73.4
	180	84.3	84.4

^a^Significant differences are observed between the levels of H_2_O_2_-induced DNA damage in BC patients and healthy controls (*p* = 0.00035, 0.045, 0.037, and 0.026 at 0, 30, 60, and 180 min after mutagenic exposure according to two sided Student's *t*-test, and 0.01 < *p* < 0.05 according to the nonparametric Mann-Whitney *U* test).

**Table 4:** The cellular response to DNA damage in the groups of BC patients, elderly people, and individuals with chronic inflammatory diseases.

Features under study	BC patients (*n* = 40)	Individuals older than 60 years (*n* = 15)	Individuals with chronic inflammations (*n* = 15)
Average age (mean ± SE)	69.55 ± 1.57^a^	62.8 ± 0.74	48.87 ± 2.86
Sex ratio females/males (% of males)	6/34 (85)	4/11 (73.33)	7/8 (53.33)
Smokers/nonsmokers (% of smokers)	5/35 (89)	6/9 (60)	2/13 (13.33)
Basal DNA damage (a.u.) at 180 min	11.38 ± 1.09	9.93 ± 2.61	6.0 ± 1.32
H_2_O_2_-induced DNA damage (a.u.) at 0 min	117.13 ± 7.01^b^	89.33 ± 11.55	92.47 ± 7.97
Residual level of H_2_O_2_-induced DNA damage (a.u.) at 180 min	17.7 ± 1.59	18.2 ± 4.21	12.77 ± 3.03
DNA repair efficiency for 30 min incubation	65.24 ± 2.08	69.91 ± 3.82	66.23 ± 4.03
DNA repair efficiency for 180 min incubation	84.25 ± 1.33	81.27 ± 3.27	84.72 ± 3.22

^a^Significant differences concerning age were revealed between BC patients and elderly persons (*p* = 0.0004) and between BC patients and individuals with chronic inflammatory diseases (*p* = 0.0001).

^b^Significant differences concerning the initial level of H_2_O_2_-induced DNA damage were observed between BC patients and elderly persons (*p* = 0.05) and between those and individuals with inflammations (*p* = 0.027).

**Table 5:** The frequency of individuals with increased lymphocyte sensitivity to DNA damage in various study groups.

Study groups	Proportion of sensitive subjects (%) with respect to			
	Basal DNA damage	H_2_O_2_-induced DNA damage		Total
		Initial level	Residual level	
BC patients (*n* = 40)	17.5	50^a^	10	62.5^b^
Individuals older than 60 years (*n* = 15)	20.0	26.67	26.67	46.67
Individuals with chronic inflammatory diseases (*n* = 15)	6.67	26.67	6.67	46.67
Controls (*n* = 35)	8.57	14.29	5.71	40.0

^a^Significant differences were revealed between all the groups by criterion *χ* ^2^ (*p* = 0.009) and between the case group and controls (*p* = 0.001).

^b^Significant differences were observed between BC patients and controls (*p* = 0.05).

**Table 6:** Distribution of allelic variants of some DNA repair genes in the group of BC patients as compared to controls.

Genotypes/variant alleles	BC cases		Controls		*p*
	*n*	%	*n*	%	
*ERCC2/XPD* Lys751Gln (rs13181)					
Lys/Lys	120	29.2	132	36.2^a^	0.039
Lys/Gln	212	51.6	160	43.8^b^	0.031
Gln/Gln	79	19.2	73	20.0	>0.05
Lys/Gln + Gln/Gln	291	70.8	233	63.8^c^	0.039
Gln	370/822	45.0	306/730	41.9	>0.05
*ERCC6/CSB* Gly399Asp (rs2228528)					
Gly/Gly	283	68.0	259	71.0	>0.05
Gly/Asp	121	29.1	101	27.7	>0.05
Asp/Asp	12	2.9	5	1.4	>0.05
Gly/Asp + Asp/Asp	133	32.0	106	29.0	>0.05
Asp	145/832	17.4	111/730	15.2	>0.05

The genotypic distribution is in accordance with Hardy-Weinberg equilibrium in the control and case groups: *χ* ^2^ = 3.63 and 0.72 (*p* = 0.06 and 0.39) for *ERCC2/XPD* Lys751Gln polymorphism; *χ* ^2^ = 1.95 and 0.05 (*p* = 0.16 and 0.83) for *ERCC6/CSB* Gly399Asp polymorphism.

^a^OR [95% CI] = 0.73 [0.54–0.98], *p* = 0.039; ^b^OR [95% CI] = 1.36 [1.03–1.81], *p* = 0.031; ^c^OR [95% CI] = 1.37 [1.02–1.86], *p* = 0.039. OR values describe a homozygous wild type genotype of the *XPD* gene as a protective factor, whereas the heterozygous genotype and sum of genotypes containing a variant allele seem to be risk factors for developing bladder cancer.

**Table 7:** Distribution of genotypes of some DNA repair genes in non-muscle-invasive (Ta/T1) and muscle-invasive (T ≥ 2) tumors depending on their differentiation.

DNA repair gene polymorphisms	Genotype frequency (%) depending on the tumor stages and grades						
	Ta/T1	T ≥ 2	G1	G2	G3	low	high
*OGG1* 326 rs1052133	*n* = 288	*n* = 126	*n* = 135	*n* = 185	*n* = 86	*n* = 240	*n* = 156
Ser/Ser	67.0	69.0	71.1	65.9	67.4	67.5	69.2
Ser/Cys	28.8	27.0	24.4	30.8	27.9	28.8	26.3
Cys/Cys	4.2	4.0	4.4	3.2	4.7	3.8	4.5

*XRCC1* 399 rs25487	*n* = 288	*n* = 126	*n* = 135	*n* = 186	*n* = 86	*n* = 241	*n* = 156
Arg/Arg	39.6	44.4	41.5	38.2	46.5	39.0	44.2
Arg/Gln	49.0	44.4	48.9	48.4	43.0	48.5	44.9
Gln/Gln	11.5	11.1	9.6	13.4	10.5	12.5	10.9

*XPD *312 rs1799793	*n* = 288	*n* = 126	*n* = 135	*n* = 185	*n* = 86	*n* = 240	*n* = 156
Asp/Asp	29.2	32.5	24.4	35.7	30.2	31.3	30.8
Asp/Asn	54.5	49.2	62.2	44.9	52.3	53.7	49.3
Asn/Asn	16.3	18.3	13.3	19.5	17.5	15.0	19.9

*XPD *751 rs13181	*n* = 280	*n* = 124	*n* = 131	*n* = 181	*n* = 84	*n* = 234	*n* = 152
Lys/Lys	28.9	30.6	28.2	30.4	31.0	31.2	28.3
Lys/Gln	53.2	51.6	57.3	49.2	51.2	52.1	52.0
Gln/Gln	17.9	17.7	14.5	20.4	17.8	16.7	19.7

*ERCC6* 1097 rs2228526	*n* = 289	*n* = 126	*n* = 135	*n* = 186	*n* = 86	*n* = 241	*n* = 156
Met/Met	50.9	46.8	57.0	45.2	47.7	48.1	49.3
Met/Val	44.3	40.5	37.8	47.3	43.0	45.6	41.7
Val/Val	4.8	12.7^a^	5.2	7.5	9.3	6.2	9.0

*ERCC6* 399 rs2228528	*n* = 283	*n* = 126	*n* = 133	*n* = 183	*n* = 85	*n* = 236	*n* = 155
Gly/Gly	67.8	67.5	65.4	69.4	67.0	65.7	69.7
Gly/Asp	29.0	30.2	31.6	27.3	30.6	31.4	27.7
Asp/Asp	3.2	2.4	3.0	3.3	2.4	2.9	2.6

^a^Significant differences were observed between Ta/T1 and T ≥ 2 tumors with respect to frequencies of the homozygous *ERCC6 *1097 Val/Val genotype according to *χ* ^2^ test (*p* = 0.0045).
